# Gut microbiota regulates host melatonin production through epithelial cell MyD88

**DOI:** 10.1080/19490976.2024.2313769

**Published:** 2024-02-14

**Authors:** Bingnan Liu, Lijuan Fan, Youxia Wang, Hao Wang, Yuqi Yan, Shuai Chen, Ifen Hung, Chunxue Liu, Hong Wei, Liangpeng Ge, Wenkai Ren

**Affiliations:** a State Key Laboratory of Swine and Poultry Breeding Industry, College of Animal Science, South China Agricultural University, Guangzhou, China; b National Center of Technology Innovation for Pigs, Chongqing, China; c Department of Veterinary Medicine, College of Animal Sciences, Zhejiang University, Hangzhou, China; d Key Laboratory of Systems Health Science of Zhejiang Province, School of Life Science, Hangzhou Institute for Advanced Study, University of Chinese Academy of Sciences, Hangzhou, China; e Anyou Biotechnology Group Co. LTD, Taicang, China; f Joint Laboratory of Functional Nutrition and Animal Health, Centree Bio-tech (Wuhan) Co., LTD, Wuhan, China; g State Key Laboratory of Agricultural Microbiology, College of Animal Sciences and Technology, Key Laboratory of Agricultural Animal Genetics, Breeding, and Reproduction of the Ministry of Education & Key Laboratory of Swine Genetics and Breeding of Ministry of Agriculture and Rural Affairs, Huazhong Agricultural University, Wuhan, China; h Chongqing Academy of Animal Sciences, Key Laboratory of Pig Industry Science, Ministry of Agriculture, Chongqing, China

**Keywords:** Melatonin, lactobacillus reuteri, Escherichia coli, MyD88, AANAT

## Abstract

Melatonin has various physiological effects, such as the maintenance of circadian rhythms, anti-inflammatory functions, and regulation of intestinal barriers. The regulatory functions of melatonin in gut microbiota remodeling have also been well clarified; however, the role of gut microbiota in regulating host melatonin production remains poorly understood. To address this, we studied the contribution of gut microbiota to host melatonin production using gut microbiota-perturbed models. We demonstrated that antibiotic-treated and germ-free mice possessed diminished melatonin levels in the serum and elevated melatonin levels in the colon. The influence of the intestinal microbiota on host melatonin production was further confirmed by fecal microbiota transplantation. Notably, *Lactobacillus reuteri* (*L. R*) and *Escherichia coli* (*E. coli*) recapitulated the effects of gut microbiota on host melatonin production. Mechanistically, *L. R* and *E. coli* activated the TLR2/4/MyD88/NF-κB signaling pathway to promote expression of arylalkylamine N-acetyltransferase (AANAT, a rate-limiting enzyme for melatonin production), and MyD88 deficiency in colonic epithelial cells abolished the influence of intestinal microbiota on colonic melatonin production. Collectively, we revealed a specific underlying mechanism of gut microbiota to modulate host melatonin production, which might provide novel therapeutic ideas for melatonin-related diseases.

## Introduction

Melatonin is generated from tryptophan (Trp) via the 5-hydroxytryptamine (5-HT) pathway, in which arylalkylamine N-acetyltransferase (AANAT) functions as a rate-limiting enzyme. Melatonin is produced in the pineal gland and other peripheral organs and has various physiological functions, including sleep initiation, anti-aging, and anti-inflammatory properties,^1–3^ and its deficiency may lead to various diseases. For example, melatonin deficiency caused by sleep deprivation results in cognitive impairments and intestinal barrier dysfunction,^4,5^ and *Aanat* deficiency-induced melatonin reduction is involved in the pathogenesis of Alzheimer’s disease and obesity in mice.^6^ Thus, maintaining sufficient melatonin production is crucial for health maintenance.

Melatonin synthesis is closely related to the expression of AANAT, and intrinsic mechanisms in regulation of AANAT expression have been described.^7,8^ The initiation of *Aanat* transcription is controlled by the norepinephrine-stimulated phosphorylation of cAMP response element-binding protein (p-CREB), as the p-CREB binds to CRE in the promoter of *Aanat* to initiate transcription.^9^ Besides, extrinsic factors are also involved in melatonin production. For instance, melatonin synthesis in the pineal gland follows a clear circadian rhythm,^10^ while melatonin production in the gastrointestinal tract could be regulated by the composition of food ingested.^11,12^ Although these factors have been well clarified, the role of gut microbiota in regulating host melatonin production remains poorly understood. Gut microbiota converts Trp into indole and its derivatives, and the role of gut microbiota in regulating the 5-HT pathway has been revealed.^13^ Notably, gut microbiota regulates 5-HT synthesis in the serum and TPH-1 expression in the colon.^14,15^ Given that melatonin is a downstream metabolite of the Trp-5-HT pathway, we hypothesized that gut microbiota might affect host melatonin production.

Here, we found that melatonin levels decreased in the serum and increased in the colon of metronidazole-treated and germ-free (GF) mice. The influence of intestinal microbiota on host melatonin production was further confirmed by fecal microbiota transplantation. Notably, *Lactobacillus reuteri* (*L. R*) and *Escherichia coli* (*E. coli*) recapitulated the effects of gut microbiota on host melatonin production. Mechanistically, *L. R* and *E. coli* activated the TLR2/4/MyD88/NF-κB signaling pathway to promote AANAT expression, which supports melatonin production in the colon. MyD88 deficiency in the colonic epithelial cells abolished the influence of intestinal microbiota on colonic melatonin production. Overall, we revealed the underlying mechanism of gut microbiota in regulating host melatonin production, which might provide insights for melatonin-based therapeutics.

## Results

### Gut microbiota is involved in host melatonin production

**Figure 1.**
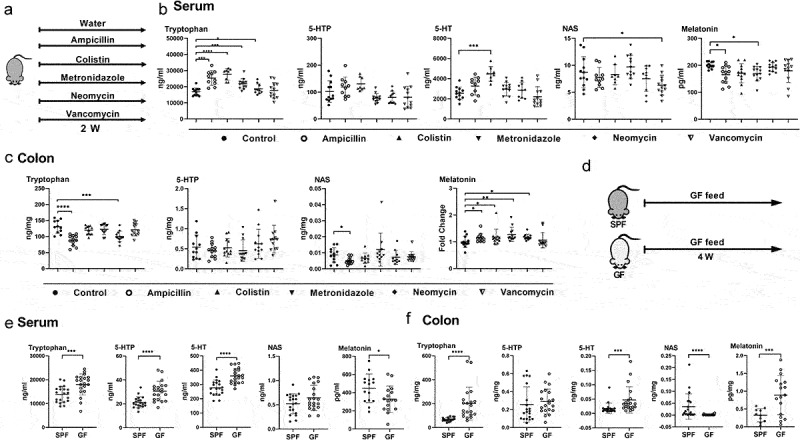
Gut microbiota is involved in host melatonin production.

Gut microbiota can influence host Trp metabolism,^16^ but whether gut microbiota affects host melatonin production is unknown. In order to deplete gut microbiota and exclude the specificity of antibiotics, we selected different kinds of broad-spectrum antibiotics (ampicillin, colistin, metronidazole, neomycin, and vancomycin) for gut microbiota depletion according to previous studies.^17,18^ We treated mice with these antibiotics and measured levels of metabolites in the Trp-5-HT pathway, including Trp, 5-hydroxytryptophan (5-HTP), 5-HT, N-acetylserotonin (NAS), and melatonin by targeted metabolomics (Figure 1a). The metabolism of Trp-5-HT pathway was affected in mice treated with different antibiotics. Notably, ampicillin and metronidazole decreased serum melatonin levels (Figure 1b), whereas ampicillin, colistin, metronidazole, and neomycin increased colonic melatonin levels (Figure 1c). However, no significant change in melatonin levels was observed in the feces of metronidazole-treated mice (Figure S1e). These results suggest that antibiotic administrations result in diverse melatonin changes in mouse serum and colon.

(a) Diagram for the description of antibiotic-treated mouse model. (b) The level of tryptophan, 5-HTP, 5-HT, NAS, and melatonin in the serum of control and antibiotic-treated mice (n = 8-12). (c) The level of tryptophan, 5-HTP, NAS, and melatonin in the colon of control and antibiotic-treated mice (n = 10-12). Melatonin levels were detected in batches and normalized by external standard. (d) Diagram for the description of treatment in SPF mice and GF mice. (e) The level of tryptophan, 5-HTP, 5-HT, NAS, and melatonin in the serum of SPF and GF mice (n = 15-20). (f) The level of tryptophan, 5-HTP, 5-HT, NAS, and melatonin in the colon of SPF and GF mice (n = 10-20). Data were analyzed by one-way ANOVA (b and c: Tryptophan, 5-HTP), or Kruskal-Wallis (c: NAS, Melatonin) or unpaired t test (e and f: 5-HTP, Melatonin), or Mann-Whitney U test (f: Tryptophan, 5-HT, NAS) and represented as mean ± SD. *p < .05, **p < .01, ***p < .001 and ****p < .0001.

To further validate the effects of gut microbiota on melatonin levels, we measured levels of metabolites in the Trp-5-HT pathway in germ-free (GF) mice (Figure 1d). Similar to mice treated with antibiotics, metabolism of Trp-5-HT pathway was also affected in the serum and colon of GF mice, like lower level of serum melatonin and higher level of colonic melatonin (Figure 1e–f). Collectively, these results suggest that melatonin production is altered in the serum and colon of GF mice.

### Melatonin production is regulated by fecal microbiota transplantation

**Figure 2.**
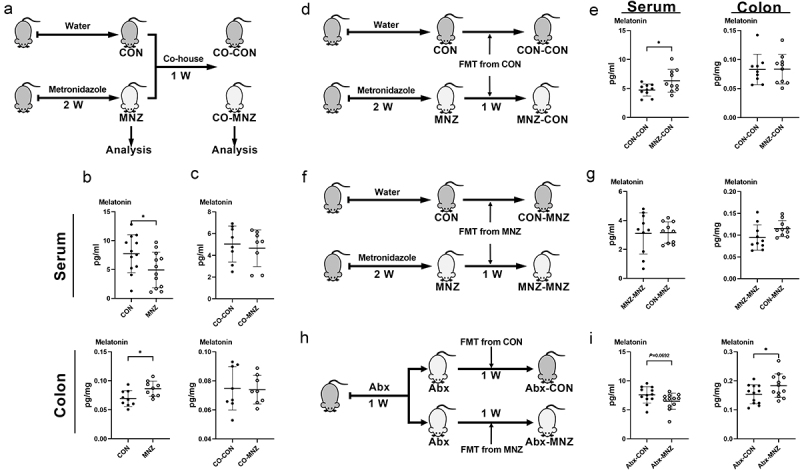
Melatonin production is regulated by fecal microbiota transplantation.

To further validate the influence of gut microbiota on melatonin production, we co-housed mice that were pretreated and not pretreated with metronidazole (Figure 2a). Although metronidazole administration decreased serum melatonin and elevated colonic melatonin (Figure 2b), the two groups of co-housed mice showed comparable melatonin levels in both the serum and colon (Figure 2c). We then investigated whether fecal microbiota transplantation (FMT) could alter melatonin levels in mice. FMT from the control group resulted in increased serum melatonin levels in mice pretreated with metronidazole, as well as comparable colonic melatonin levels between the control and metronidazole-pretreated groups of mice (Figure 2d–e). After receiving FMT from the metronidazole-pretreated group, alterations in melatonin levels in the serum or colon in both groups of mice that were previously detected were eliminated (Figure 2f–g). Notably, mice that were pretreated with a cocktail of antibiotics (Abx) received FMT from control mice and metronidazole-treated mice, respectively, and showed similar patterns of melatonin level alterations as their donor mice (Figure 2h–i). Overall, these results suggest that melatonin alterations in the serum and colon of mice is regulated by intestinal microbiota.

(a) Diagram for the description of co-housed mouse model. (b) The level of melatonin in the serum and colon of control (CON) and metronidazole-treated (MNZ) mice (n = 9-12). (c) The level of melatonin in the serum and colon of CON and MNZ mice after co-housing (n = 8). (d) Diagram for the description of CON FMT in CON and MNZ mice. (e) The level of melatonin in the serum and colon of CON and MNZ mice with CON FMT (n = 9-10). (f) Diagram for the description of MNZ FMT in CON and MNZ mice. (g) The level of melatonin in the serum and colon of CON and MNZ mice with MNZ FMT (n = 10). (h) Diagram for the description of FMT in Abx cocktail pretreated mice. (i) The level of melatonin in the serum and colon of Abx cocktail pretreated mice with FMT from CON or MNZ mice (n = 12). Data were analyzed by unpaired t test (b-c, e, g, i) and represented as mean ± SD. *p <.05. FMT, fecal microbiota transplantation; Abx, antibiotics.

### Lactobacillus reuteri and Escherichia coli are involved in host melatonin production

To characterize how metronidazole-induced alterations in bacteria are related to melatonin production in mice, we performed 16S rDNA sequencing of fecal bacterial DNA collected from metronidazole-treated mice to obtain microbial species richness. In LEfSe analysis, various bacterial species showed changes in mice treated with metronidazole (Figure S2a). Next, Spearman’s rank correlation was performed to characterize the relationship between microbial species richness and melatonin levels in the serum and colon, respectively. Various bacterial species were associated with serum and colonic melatonin levels, among which *Lactobacilli* including *Lactobacillus murinus* (*L. M*), *Lactobacillus intestinalis* (*L. I*), *Lactobacillus johnsonii* (*L. J*) *and Lactobacillus reuteri* (*L. R*), were positively correlated with serum melatonin levels but negatively correlated with colonic melatonin levels (Figure S2b-c). The abundance of *E. coli* was negatively correlated with serum melatonin levels (Figure S2B). Notably, the abundance of *L. M, L. I, L. J* and *L. R* were decreased but *E. coli* was increased in mice treated with metronidazole (Figure S2d-e). Previous studies have shown that *Lactobacilli* promotes melatonin production, and *Lactobacillus rhamnosus* enhances the abundance of melatonin receptors,^19,20^ suggesting that *Lactobacilli* may regulate host melatonin production. In addition, a correlation between *E. coli* and melatonin production in host cells has also observed.^21–23^ These results suggest that *L. M, L. I, L. J, L. R* or *E. coli* may regulate host melatonin production.

**Figure 3.**
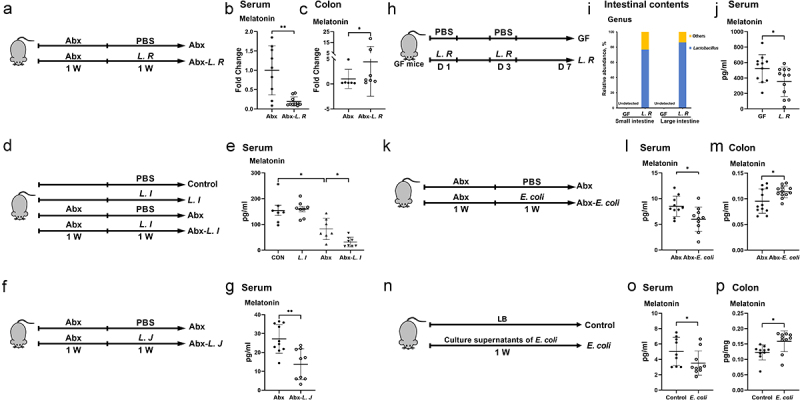
*L. R*, *L. I*, *L. J* or *E. coli* colonization affects melatonin production *in vivo*.

To further determine their roles in modulating melatonin production, strains of *L. M*, *L. R*, *L. I, L. J* or *E. coli* were individually colonized into our previous established pseudo-GF (antibiotic cocktail pretreated) model, in which an antibiotic cocktail containing ampicillin, metronidazole, neomycin, and vancomycin was used for maximal microbiome depletion.^24,25^ In antibiotic cocktail-pretreated mice, *L. R* colonization decreased serum melatonin levels and increased colonic melatonin levels (Figure 3a–c). Although had little effect on serum melatonin levels in the control group, *L. I* colonization decreased serum melatonin levels in the Abx cocktail-pretreated group (Figure 3d–e). In addition, *L. J* colonization decreased serum melatonin levels in Abx cocktail-pretreated mice (Figure 3f–g). In order to further verify the effects of *L. R* on host melatonin production, *L. R* was colonized into GF mice. The relative abundance of *Lactobacillus* in both the small and large intestines of GF mice was much higher than that in the control group (undetected), and *L. R* colonization decreased serum melatonin levels (Figure 3h–j). However, the change in melatonin production induced by *L. R*, *L. I* or *L. J* colonization was inconsistent with the above spearman’s rank correlation analysis (Figure S2b-c), which may be related to the regulation of post-antibiotic gut mucosal composition, function and microbiome reconstitution by these strains.^26,27^ In Abx cocktail-pretreated mice, *E. coli* colonization decreased serum melatonin levels and increased colonic melatonin levels (Figure 3k–m), whereas *L. M* colonization did not affect host melatonin production (Figure S3a-c), indicating the strain specific regulatory function in melatonin production. Collectively, these results suggest that *L. R*, *L. I*, *L. J* or *E. coli* colonization affects host melatonin production in the serum and colon.

(a) Diagram for the description of *L. R* colonization in Abx cocktail pretreated mice. (b and c) The level of melatonin in the serum (b) and colon (c) of Abx cocktail pretreated micecolonized with *L. R* or not (*n* = 6-10). Melatonin levels were detected in batches and normalized by external standard. (d) Diagram for the description of *L. I* colonization in SPF and Abx cocktail pretreated mice. (e) The level of melatonin in the serum of SPF and Abx cocktail pretreated mice colonized with *L. I* or not (*n* = 7-8). (f) Diagram for the description of *L. J* colonization in Abx cocktail pretreated mice. (g) The level of melatonin in the serum of Abx cocktail pretreated mice colonized with *L. J* or not (*n* = 9-10). (h) Diagram for the description of *L. R* colonization in GF mice. (i) Relative abundance of *Lactobacillus* in the intestinal contents of GF mice colonized with *L. R* or not (*n* = 4). (j) The level of melatonin in the serum of GF mice colonized with *L. R* or not (*n* = 11-12). (k) Diagram for the description of *E. coli* colonization in Abx cocktail pretreated mice. (l and m) The level of melatonin in the serum (l) and colon (m) of Abx cocktail pretreated mice colonized with *E. coli* or not (*n* = 10-12). (n) Diagram for the description of *E. coli* culture supernatant gavage in mice. (o and p) The level of melatonin in the serum (o) and colon (p) of mice with LB broth or *E. coli* culture supernatant gavage (*n* = 9-10). Data were analyzed by unpaired *t* test (e, g, j, l-m) or Mann-Whitney *U* test (b-c, o-p) and represented as mean ± SD. *p < .05, **p < .01.

To explore the underlying molecular mechanisms of above commensal bacterial strains in host melatonin production, we cultured these bacterial strains *in vitro* and detected melatonin levels in their respective culture supernatants. Melatonin was not detected in any culture supernatant at any time point (Figure S4a-f), suggesting that alterations in melatonin levels we previously detected might be a result of interaction between gut microbes and the host. Compelling results indicate links between microbial metabolites and host metabolism,^28,29^ therefore, we hypothesized that these bacteria affect host melatonin production through their metabolites. Given that *L. R*, *L. I* and *L. J* all belong to the same bacterial taxa and have similar effects on serum melatonin production, we assumed that the common metabolite, lactate, may contribute to the effects of *L. R*, *L. I* or *L. J* on melatonin production. To test this hypothesis, we orally administrated culture supernatants of *L. R*, *L. I* or *L. J* or lactate to mice. Unlike what was observed in *L. R*, *L. I* and *L. J* colonized mice, all culture supernatants or lactate administration failed to recapitulate the effects of *L. R*, *L. I* or *L. J* colonization on serum melatonin in mice, whereas administration of *L. R* culture supernatants to mice decreased colonic melatonin levels (Figure S5a-c). Interestingly, the administration of *E. coli* culture supernatants recapitulated the effect of *E. coli* colonization in mice, which decreased serum melatonin levels and increased colonic melatonin levels (Figure 3n–p). Thus, the underlying molecular mechanisms of above commensal bacterial strains in host melatonin production need further well-designed investigations.

### Gut microbiota promotes AANAT expression through NF-κB signaling

**Figure 4.**
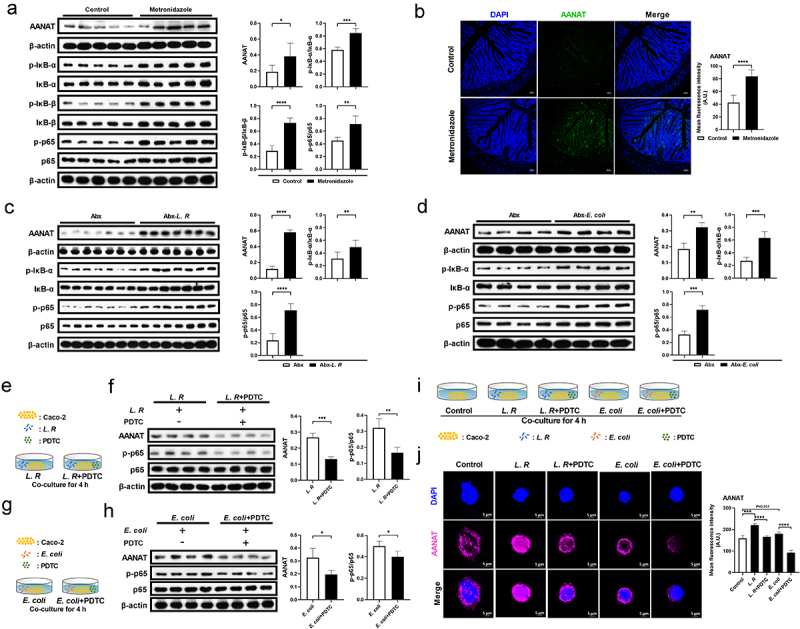
Gut microbiota regulates AANAT expression through NF-κB signaling.

AANAT is a rate-limiting enzyme for melatonin biosynthesis and exhibits alteration synchronous with melatonin level. Therefore, in this study, we detected AANAT expression in mouse models with elevated colonic melatonin levels (Figures 2a–b, 3a–c & 3k–m). Immunoblotting results showed that metronidazole administration up-regulated AANAT expression and activated nuclear factor kappa-B (NF-κB) signaling in mouse colon (Figure 4a). Immunofluorescence staining also confirmed that metronidazole administration elevated AANAT expression in the colon, particularly in colonic epithelial cells (Figure 4b). Consistently, elevated AANAT expression and NF-κB activation were observed in mice with *L. R* or *E. coli* colonization (Figure 4c–d). Subsequently, in order to verify whether the elevation of AANAT expression resulting from gut microbiota alterations depends on NF-κB signaling, Caco-2 cells that were co-cultured with *L. R* or *E. coli* were treated with a NF-κB inhibitor, pyrrolidine dithiocarbamate ammonium (PDTC) (Figures 4e, g & 4I). As expected, the inhibition of NF-κB signaling blocked the elevation of AANAT expression in Caco-2 cells co-cultured with *L. R* or *E. coli* (Figure 4f, h, j). These results indicate that intestinal microbiota promotes AANAT expression via NF-κB signaling.

(a) The protein abundance of AANAT, p-IκB-α, IκB-α, p-IκB-β, IκB-β, p-p65 and p65 in the colon of mice treated with metronidazole or not (*n* = 5). (b) Immunofluorescence analysis of AANAT (green) in the colon tissue of mice treated with metronidazole or not. Scale bars, 50 μm (*n* = 6). (c) The protein abundance of AANAT, p-IκB-α, IκB-α, p-p65 and p65 in the colon of Abx cocktail pretreated mice colonized with *L. R* or not (*n* = 7). (d) The protein abundance of AANAT, p-IκB-α, IκB-α, p-p65 and p65 in the colon of Abx cocktail pretreated mice colonized with *E. coli* or not (*n* = 4). (e) Diagram for the description of Caco-2 cell co-culture system with *L. R* and PDTC. (f) The protein abundance of AANAT, p-p65 and p65 in*L. R* co-cultured Caco-2 cells treated with PDTC or not (*n* = 4). (g) Diagram for the description of Caco-2 cell co-culture system with *E. coli* and PDTC. (h) The protein abundance of AANAT, p-p65 and p65 in *E. coli* co-cultured Caco-2 cells treated with PDTC or not (*n* = 4). (i) Diagram for the description of Caco-2 cell co-culture system with *L. R*/*E. coli* and PDTC. (j) Immunofluorescence analysis of AANAT (purple) in *L. R* or *E. coli* co-cultured Caco-2 cells treated with PDTC or not. Scale bars, 5 μm (*n* = 3-5). Data were analyzed by unpaired *t* test (a-d, f, h, j) and represented as mean ± SD. *p < .05, **p < .01, ***p < .001 and ****p < .0001.

### Myd88 deficiency in intestinal epithelial cells blocks the effects of gut microbiota on host melatonin production

**Figure 5.**
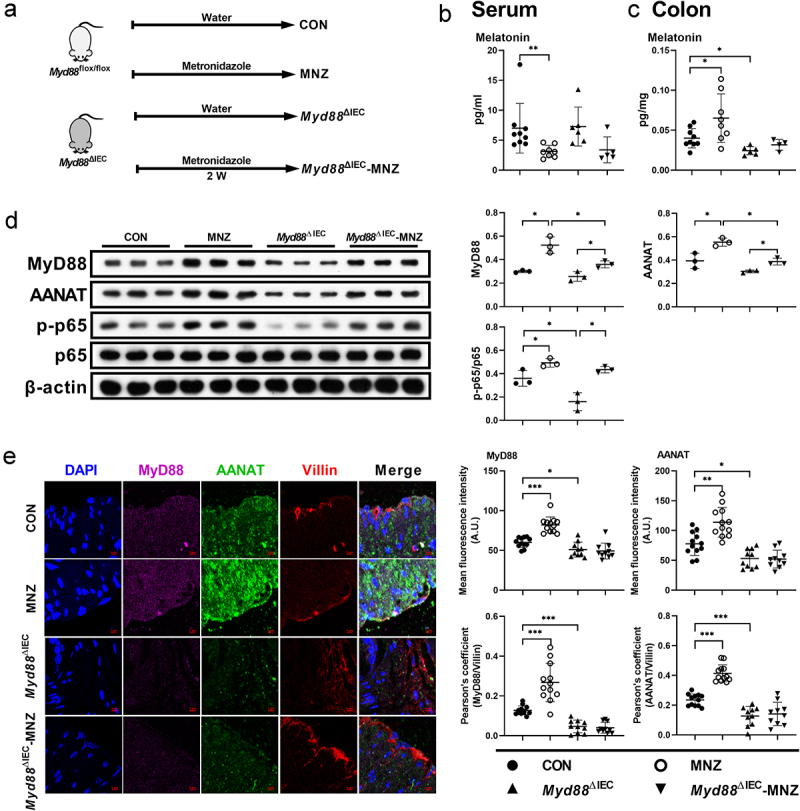
MyD88 deficiency eliminates the effects of metronidazole treatment on melatonin production.

**Figure 6.**
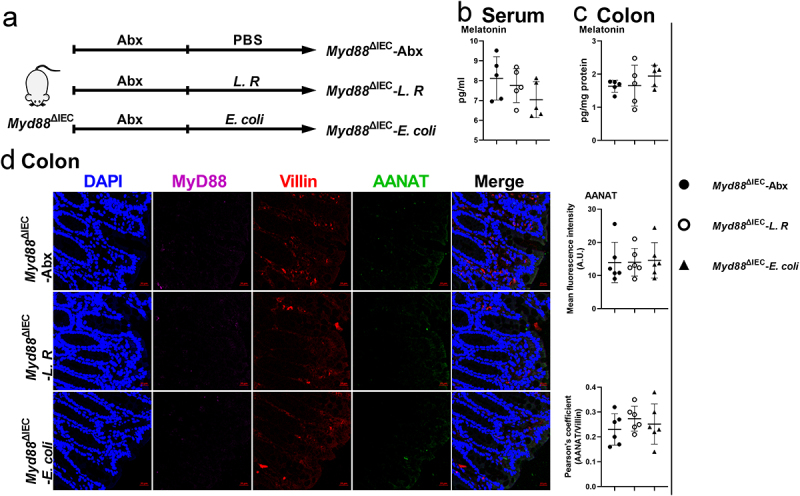
MyD88 deficiency eliminates the effects of *L. R* or *E. coli* colonization on melatonin production.

We then investigated how gut microbiota activates NF-κB signaling in colonic epithelial cells. Given that gut microbiota activates NF-κB signaling, which functions downstream of MyD88, we crossed *Myd88*^*flox/flox*^ mice with Villin-Cre mice to generate intestinal epithelial *Myd88* depleted (*Myd88*^ΔIEC^) mice (Figure S6a-b). Notably, there was no difference in serum or colonic melatonin levels observed in *Myd88*^ΔIEC^ mice administered with metronidazole, which was not the case for *Myd88*^*flox/flox*^ mice (Figure 5a–c). However, we still observed an increased abundance of MyD88, AANAT, and the ratio of p-p65/p65 in the colon of metronidazole-treated *Myd88*^ΔIEC^ mice (Figure 5d), which might be due to the use of entire colonic tissue in immunoblotting. To rule out the involvement of other cell types, we colocalized MyD88 and AANAT with Villin (a marker of intestinal epithelial cells) and found *Myd88*^ΔIEC^ mice, regardless of whether metronidazole treated, showed a comparable abundance of MyD88 and AANAT, which were Villin colocalized in colonic epithelial cells (Figure 5e). These results suggest that colonic epithelial cell MyD88 signaling is required for AANAT elevation induced by metronidazole treatment. MyD88 deficiency in mouse colonic epithelial cells eliminated serum and colonic melatonin level alterations induced by *L. R* or *E. coli* colonization (Figure 6a–c). In addition, *L. R* or *E. coli* colonization had no effect on expression of AANAT colocalized with Villin in *Myd88*^ΔIEC^ mice (Figure 6d). Taken together, MyD88 deficiency in colonic epithelial cells blocks the effects of gut microbiota on host melatonin production.

(a) Diagram for the description of metronidazole treatment in *Myd88^flox/flox^* and *Myd88*^ΔIEC^ mice. (b and c) The level of melatonin in the serum (b) and colon (c) of *Myd88^flox/flox^* and *Myd88*^ΔIEC^ mice treated with metronidazole or not (*n* = 4-10). (d) The protein abundance of MyD88, AANAT, p-p65 and p65 in the colon of *Myd88^flox/flox^* and *Myd88*^ΔIEC^ mice treated with metronidazole or not (*n* = 3). (e) Confocal microscopy analysis of MyD88 (purple), AANAT (green), and Villin (red) in the colon tissue of *Myd88^flox/flox^* and *Myd88*^ΔIEC^ mice treated with metronidazole or not. Scale bars, 5 μm (*n* = 10-12). Data were analyzed by Kruskal-Wallis (b) or one-way ANOVA (c-e) and represented as mean ± SD. *p < .05, **p < .01, ***p < .001.

(a) Diagram for the description of *L. R* or*E. coli* colonization in *Myd88*^ΔIEC^ mice. (b and c) The level of melatonin in the serum (b) and colon (c) of *Myd88*^ΔIEC^ mice colonized with *L. R/E. coli* or not (*n* = 5). (d) Confocal microscopy analysis of MyD88 (purple), AANAT (green) and Villin (red) in the colon tissue of *Myd88*^ΔIEC^ mice colonized with *L. R/E. coli* (Scale bars, 20 μm, *n* = 6). Data were analyzed by one-way ANOVA (b-d) and represented as mean ± SD.

### Gut microbiota activates the NF-κB pathway through TLR2 and TLR4 signaling

**Figure 7.**
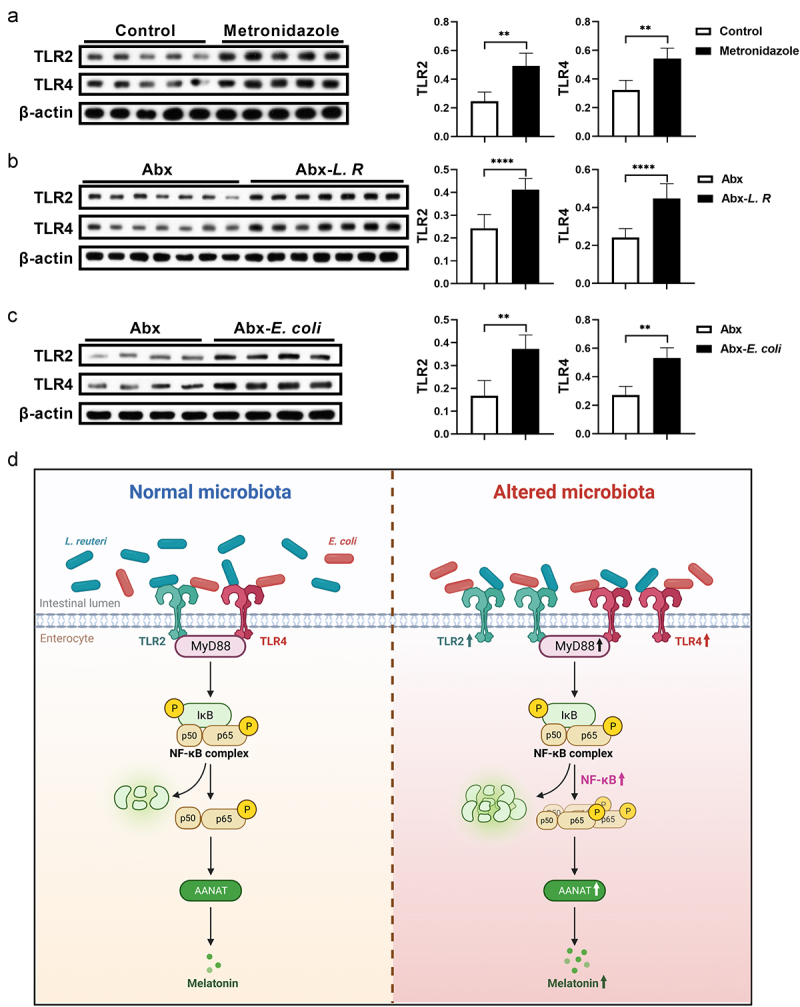
Gut microbiota activates TLR2 and TLR4 signaling in the colon.

Given that MyD88 adapter proteins link members of the toll-like receptors (TLRs), such as TLR2 and TLR4, to activate NF-κB,^30^ we analyzed abundance of TLR2 and TLR4 in mouse colon with altered melatonin levels (Figures 2a–b, 3a–c & 3k–m). Interestingly, both TLR2 and TLR4 were significantly increased in the colon of metronidazole-treated and *L. R*/*E. coli* colonized mice (Figure 7a–c). Taken together, these results revealed that gut microbiota regulates host melatonin production via TLR2 and TLR4 signaling, which further elevates AANAT expression by activating the MyD88/NF-κB signaling pathway (Figure 7d).

(a) The protein abundance of TLR2 and TLR4 in the colon of mice treated with metronidazole or not (*n* = 5). (b) The protein abundance of TLR2 and TLR4 in the colon of Abx cocktail pretreated mice colonized with *L. R* or not (*n* = 7). (c) The protein abundance of TLR2 and TLR4 in the colon of Abx cocktail pretreated mice colonized with *E. coli* or not (*n* = 4). (d) Diagram depicting the mechanisms of gut microbiota regulating melatonin production in colonic epithelial cells. The diagram was created with BioRender.com. Data were analyzed by unpaired *t* test (a-c) and represented as mean ± SD. **p < .01 and ****p < .0001.

## Discussion

Melatonin exhibits antioxidative, anti-inflammatory, and anti-infective properties by remodeling gut microbiomes.^2,3,24^ However, little attention has been paid to the regulation of gut microbiota on host melatonin production. In this study, we observed decreased levels of serum melatonin and increased levels of colonic melatonin in metronidazole-treated and GF mice, suggesting that gut microbiota regulates host melatonin production. Interestingly, the gut microbiota induced changes of melatonin levels are different in the serum and colon. It is known that melatonin in the blood is mainly synthesized and released by the pineal gland, while peripheral organs, including gastrointestinal tract, retina and skin, can also synthesize melatonin.^31^ The secretion of melatonin in the pineal gland is inhibited by NF-κB signaling,^32^ while activation of NF-κB signaling stimulates immunocompetent cells to promote melatonin production,^23^ suggesting that the opposite change of melatonin levels in the serum and colon may be due to the bidirectional regulation of NF-κB signaling on pineal gland and colon. However, the underlying mechanism for this difference needs more well-designed investigations. Notably, we only investigated serum and colonic melatonin level alterations caused by gut microbiota clearance, whether gut microbiota regulates melatonin production in other tissues requires further exploration.

Gut microbiota is associated with host metabolism of energy, lipids, and glucose.^33^ For instance, *Klebsiella pneumoniae* sequence type 258 (Kp ST258) induces host glutaminolysis and fatty acid oxidation,^34^ *Bifidobacterium longum_MG723* or *Bifidobacterium bifidum_MG731* improves glucose homeostasis in diet-induced obese mice,^35^ and *Clostridium difficile* promotes the production of sorbitol.^36^ Here, *L. R* or *E. coli* colonization results in decreased melatonin levels in the serum and increased melatonin levels in the colon of Abx cocktail pretreated mice. Although we observed altered melatonin levels in mice treated with ampicillin, we did not identify bacteria that could potentially regulate melatonin production. Since antibacterial spectra are different for antibiotics, other bacteria in the gut might also affect host melatonin production. For instance, *Roseburia hominis* increases intestinal melatonin levels via its metabolites, propionate and butyrate.^37^ Furthermore, we only used antibiotics to alter the composition of intestinal bacteria; the roles of fungi and viruses in host metabolism regulation, however, cannot be ignored,^38,39^ and whether they are also involved in the regulation of host melatonin production requires further investigation. Therefore, fully exploring and evaluating the potential effects of gut microbiota on host melatonin production might be a potential direction for the targeted regulation of melatonin homeostasis.

Microbial metabolites, such as short-chain fatty acids (SCFAs), indole-3-pyruvic acid (IPA), and trimethylamine (TMA), can be sensed by the intestinal epithelium and orchestrate host physiology or diseases.^40,41^ For example, *Akkermansia muciniphila* ameliorates metabolic disorders by regulating genes related to intestinal barrier function via TLRs.^42–44^ In this study, administration of *E. coli* culture supernatants decreases the level of serum melatonin and elevates its colonic level, which is similar to the effect of *E. coli* colonization, but whether they function through the same mechanism is still unclear. Interestingly, we observed that *L. R* colonization increases colonic melatonin levels in mice, while oral administration of *L. R* culture supernatants decreases colonic melatonin levels in mice. Thus, oral administration of *L. R* culture supernatants may have different mechanisms to regulate colonic melatonin production, compared to *L. R* colonization. After the colonization of *L. R*, the composition of intestinal microbiota is changed, in which *L. R* occupies an important niche and increases colonic melatonin levels by activating the TLR2/4/MyD88/NF-κB signaling pathway. It is known that bacterial culture supernatants contain bacterial metabolites which affect the microbial community. For example, the culture supernatants of *Clostridium difficile* impact common gut microbiota associated with colonization resistance,^45^ and the culture supernatants of *Bacteroides* directly limits the growth of *Salmonella typhimurium*.^46^ However, the mechanism by which *L. R* supernatants regulate intestinal microbial composition and colonic melatonin production needs further investigation. Indeed, gut microbiota also influences host metabolism through bacterial derivatives such as microRNA, extracellular vesicles, and membrane proteins.^47,48^ For example, gut microbial DNA-containing extracellular vesicles (mEVs) are delivered to the metabolic tissues in obesity, and the leakage of mEVs contributes to the development of obesity-associated inflammation and metabolic diseases.^49^ Unfortunately, we did not reveal how *L. R* activates TLR2/4/MyD88/NF-κB signaling, but the potential of *L. R* as a probiotic in regulating melatonin production is worthy of further exploration.

Melatonin is involved in the pathogenesis of many diseases.^50–52^ Importantly, melatonin has an anti-inflammatory effect by affecting functions of immune cells, such as T cells, B cells, and macrophages.^53–56^ For example, melatonin blocks the differentiation of pathogenic Th17 cells and boosts the generation of protective Tr1 cells in mice with multiple sclerosis.^57^ Moreover, melatonin shows anti-infective activities in inhibiting Gram-negative pathogens by targeting citrate synthase^50,58^ and fighting against coronaviruses *in vitro* at pharmacological concentrations.^59^ Mechanisms through which melatonin exhibits anti-cancer effects have been well explained, including the inhibition of proliferation, invasion, metastasis, and angiogenesis, as well as the promotion of apoptosis and cancer immunity.^60^ However, studies on melatonin therapies mostly focus on the impact of oral melatonin administration on diseases, more attention should be paid to the regulation of autonomous synthesis of melatonin.

## Conclusions

In conclusion, the present results reveal the regulatory role of gut microbiota in host melatonin production. Mechanistically, gut microbiota activates TLR2/4/MyD88/NF-κB signaling in intestinal epithelial cells, resulting in higher AANAT expression and melatonin levels in the colon. This mechanism may provide novel insights for the regulation of autonomous synthesis of melatonin.

## Methods

### Mice

Germ-free (GF) mice were kindly provided by Prof. Hong Wei, from Huazhong Agricultural University (Wuhan, China). GF and specific-pathogen-free (SPF) BALB/c mice were bred at the Third Military Medical University (Chongqing, China). GF Kunming (KM) mice for *L. R* colonization were bred at the Laboratory Animal Center of Huazhong Agricultural University (Wuhan, China). Female ICR mice were purchased from SLAC Laboratory Animal Center (Changsha, China). C57BL/6J *Myd88*^*flox/flox*^ Villin-Cre mice were kindly provided by Prof. Bie Tan, from Hunan Agricultural University (Changsha, China). In all experiments, 6 to 8 weeks female mice were used and the mice in different groups were individually housed under a 12:12 light: dark cycle unless stated otherwise. All mouse protocols were approved by the conduction of the Laboratory Animal Ethical Commission of South China Agricultural University (Guangzhou, China).

GF BALB/c mice were housed in autoclaved sterile microisolators with positively pressured HEPA-filtered sterile air, irradiated food, and autoclaved water. SPF BALB/c mice were kept in a normal environment.

For antibiotic treatment in Figure 1a–c, ICR mice were treated with ampicillin (1 g/L), metronidazole (1 g/L), colistin (1 g/L), neomycin (1 g/L), or vancomycin (0.5 g/L) in drinking water for two weeks. For metronidazole treatment in Figure 5a–c, *Myd88*^ΔIEC^ mice were also treated with metronidazole (1 g/L) in drinking water for two weeks.

For co-housing, ICR mice pretreated with metronidazole for two weeks or not were co-housed in one cage for one week. Normal drinking water was provided during the co-housing experiments.

For fecal microbiota transplantation (FMT), recipient ICR mice were pretreated with metronidazole for two weeks or not, or pretreated with a cocktail of antibiotics containing neomycin (0.5 g/L), ampicillin (1 g/L), metronidazole (1 g/L), and vancomycin (0.5 g/L) for one week. FMT was then conducted for one week. Fecal material was collected from mice pretreated with metronidazole or not, resuspended at 100 mg/mL in sterile PBS, and administered via oral gavage (200 µL/mouse).

For *L. M*, *L. R*, *L. J* or *E. coli* colonization, recipient ICR mice were pretreated with Abx cocktail for one week and discontinued one day before bacteria colonization, and oral gavage with 200 μL of bacteria suspension (2 × 10^8^ CFU/day) for one week. Especially, GF KM mice received oral gavage with 200 μL of *L. R* suspension (2 × 10^8^ CFU/day) twice a week. For *L. I* colonization, ICR mice pretreated with Abx cocktail or not received oral gavage with 200 μL of *L. I* suspension (2 × 10^8^ CFU/day) for one week. For *L. R* or *E. coli* colonization in *Myd88*^ΔIEC^ mice, mice were pretreated with Abx cocktail for one week and discontinued for one day, followed by oral gavage with 200 μL of bacteria suspension (2 × 10^8^ CFU/day) for one week.

For bacterial culture supernatant supplement in ICR mice, *L. R*, *L. I*, *L. J* and *E. coli* were cultured at 37°C for 24 h and the culture supernatants were collected. Then mice were received the supernatants by oral gavage (200 μL/day) for one week. For *E. coli* culture supernatant gavage, mice were administrated with LB broth as the control group. For *L. R*, *L. I* and *L. J* culture supernatant gavage, the control group was administrated with MRS broth, and the lactate group was orally administrated with lactate (2.6 mol/L).

### Bacteria

In this study, *Lactobacillus murinus* (*L. M*), *Lactobacillus reuteri* (*L. R*), *Lactobacillus johnsonii* (*L. J*), and *Escherichia coli* (*E. coli*) strains were isolated from fecal samples of ICR mice. *Lactobacillus intestinalis* (*L. I*) was provided by Prof. Zhihong Sun, from Inner Mongolia Agricultural University (Inner Mongolia, China). *L. R*, *L. I* and *L. J* were cultured in MRS broth (Qingdao Rishui Bio-Technologies Co., Ltd.), *E. coli* was cultured in LB broth (Sangon Biotech).

### Caco-2 cells

Caco-2 cells were cultured in RPMI 1640 supplemented with 10% FBS at 37°C with 5% CO_2_. Caco-2 cells (1 × 10^6^ cells per well) were plated in 6-well plates and were cocultured with live *L. R* (1 × 10^8^ or *E. coli* (1 × 10^8^ for 4 h. The NF-κB inhibitor pyrrolidine dithiocarbamate ammonium (PDTC, 50 μM, MedChemExpress) was added with *L. R* or *E. coli*.

### Elisa

The melatonin measurements of serum and colon samples were performed using a Mouse Melatonin ELISA Kit (Mlbio) in Figures 1b, e, f, Figures 2 b, c, e, g, i, Figures 3 g, l, m, o, p, Figure 5 b–c, Figure 6b–c and Figure S5c. The concentrations of melatonin in the serum or colon samples were quantified using the ELISA kit according to the manufacturer’s protocol.

### Immunofluorescence staining

The primary antibodies, AANAT (17990–1-AP) was purchased from Proteintech, and MyD88 (ab28763) and Villin (ab201989) were purchased from Abcam. Immunofluorescence was performed according to our previous protocols.^61^

### Western blotting

The primary antibodies AANAT (17990–1-AP), IκB-α (10268–1-AP), IκB-β (12660–1-AP), p65 (10745–1-AP), MyD88 (23230–1-AP), TLR2 (66645–1-Ig), TLR4 (19811–1-AP), and β-actin (66009–1-Ig) were purchased from Proteintech. p-IκB (ab133462) and p-p65 (ab76302) were purchased from Abcam. p-IκB-β (#4921) was purchased from Cell Signaling Technology. Colonic tissue (25 mg) was added 300 μL radio immunoprecipitation assay (RIPA) lysis buffer (Beyotime Biotechnology) for grinding and held on ice for 10 min. The supernatants were collected after centrifugation for Western blotting. Western blotting assays of Caco-2 cells were carried out using routine procedures.

### UPLC-Orbitrap-MS/MS conditions

The levels of Trp metabolites in the serum, colon and feces of mice and bacterial supernatants were analyzed using UPLC-Orbitrap-MS/MS. The conditions have been described in our previous study.^62^ Briefly, melatonin and other Trp metabolites were separated using a Thermo Fisher Scientific UPLC system (Dionex UltiMate 3000) and a C1s Hypersil Gold column (1.9 μm, 100 mm × 2.1 mm; Thermo Scientific).

### 16S rRNA gene sequencing analysis

16S rRNA gene sequencing and analysis were conducted by a commercial company (Novogene, Co., Ltd.). Briefly, DNA form intestinal content was extracted with QIAamp DNA Stool Mini Kit (Qiagen) following manufacturer’s instructions. Ribosomal 16S rDNA V4 region was amplified with universal primers: 515 F (5’-GTGCCAGCMGCCGCGGTAA-3’) and 806 R (5’-GGACTACHVGGGTWTCTAAT-3’). PCR reactions were carried out with 15 µL of Phusion® High-Fidelity PCR Master Mix (New England Biolabs), 0.2 µM of forward and reverse primers, and 10 ng template DNA. Thermal cycling consisted of initial denaturation at 98°C for 1 min, followed by 30 cycles of denaturation at 98°C for 10 s, annealing at 50°C for 30 s, and elongation at 72°C for 30 s and 72°C for 5 min. Then, PCR products were purified with Universal DNA Purification Kit (TianGen). Sequencing libraries were generated using NEB Next® Ultra™ II FS DNA PCR-free Library Prep Kit (New England Biolabs) following manufacturer’s recommendations. The library was checked with Qubit and real-time PCR for quantification and bioanalyzer for size distribution detection. Quantified libraries were pooled and sequenced on Illumina platforms according to effective library concentration. Paired-end reads were assigned to samples based on their unique barcode and truncated by cutting off the barcode and primer sequence. Paired-end reads were merged using FLASH (V1.2.11, http://ccb.jhu.edu/software/FLASH/), and the splicing sequences were called raw tags. Quality filtering on the raw tags were performed using the fastp (Version 0.23.1) software to obtain high-quality Clean Tags. The tags were compared with the reference database (Silva database (16S/18S), https://www.arb-silva.de/; Unite Database (ITS), https://unite.ut.ee/) using UCHIME Algorithm (http://www.drive5.com/usearch/manual/uchime_algo.html) to detect chimera sequences, and then the effective tags were finally obtained. The effective tags were analyzed (by using Uparse software, version 7.0.1001), and assigned to the same OTUs with ≥ 97% similarity. For further annotation, we screened the representative sequence from each OTU. Species annotation was performed on OTUs sequences using the Mothur method and the SSUrRNA database of SILVA138 (http://www.arb-silva.de/) (with a threshold of 0.8–1). Taxonomic information was obtained, and the community composition of each sample was analyzed at various taxonomic levels of kingdom, phylum, class, order, family, genus, and species.

### Statistical analysis

For target metabolomic analysis, data in an instrument-specific format (*raw) were converted by the Xcalibur (version 3.0, Thermo Fisher) to common data format (XLS) files, which show information on metabolites, including the calculated amount, retention time, and peak areas. Melatonin levels in different experiments were normalized to those in their control groups. Data and statistical plots in this study were processed by GraphPad Prism 8.0 (GraphPad Software). All data are presented as mean ± standard deviation (SD). Statistical analysis was conducted as described previously.^24^ Data between two groups were analyzed by unpaired *t* test if the data are in Gaussian distribution and have equal variance, or by unpaired *t* test with Welch’s correction if the data are in Gaussian distribution but show unequal variance, or by non-parametric test (Mann – Whitney *U* test) if the data are not normally distributed. Multigroup were analyzed by one-way ANOVA followed by Dunnett multiple comparisons if the date were in Normal distribution and had equal variance, or else calculated by Kruskal-Wallis followed by Dunnett multiple comparisons. *p* < 0.05 was defined as significant difference. The data in figures is presented as *p < 0.05, **p < 0.01, ***p < 0.001 and ****p < 0.0001.

## Supplementary Material

Supplemental Material

## Data Availability

The raw 16S rDNA sequencing data generated in this study have been deposited in the NCBI Sequence Read Archive (https://www.ncbi.nlm.nih.gov/sra) under the accession number PRJNA1047195.
