# Supplementary material for: Gut microbiota regulates host melatonin production through epithelial cell MyD88
Source: Gut Microbes. 2024 Feb 14;16(1):2313769. doi: 10.1080/19490976.2024.2313769 (PMC10868534; doi:10.1080/19490976.2024.2313769)
Supplement: Supplemental Material [file KGMI_A_2313769_SM6154.zip › supplementary_files_2313769_1707796552333/Figure legends clean.docx]

**Fig. S1 Alterations of metabolites in tryptophan-5-HT pathway in feces of metronidazole-treated mice.**

(A) The level of tryptophan in the feces of mice treated with metronidazole or not (n=10).

(B) The level of 5-HTP in the feces of mice treated with metronidazole or not (n=10).

(C) The level of 5-HT in the feces of mice treated with metronidazole or not (n=10).

(D) The level of NAS in the feces of mice treated with metronidazole or not (n=10).

(E) The level of melatonin in the feces of mice treated with metronidazole or not (n=9-10).

Data were analyzed by unpaired *t* test (C-E) or Mann-Whitney *U* test (A-B) and represented as means ± SD. **P* < 0.05 and ****P* < 0.001.

**Fig. S2 Alterations of gut microbiota in metronidazole-treated mice.**

(A) LEfSe analysis showing bacterial taxa that are significantly different in abundance between mice treated with metronidazole (MNZ) or not (SPF).

(B) Spearman's rank correlation analysis between microbial species richness and melatonin levels in serum.

(C) Spearman's rank correlation analysis between microbial species richness and melatonin levels in colon.

(D) Relative abundance of *L. M*, *L. R*, *L. I* and *L. J* in the feces of mice treated with metronidazole or not (n=10).

(E) Relative abundance of *E. coli* in the feces of mice treated with metronidazole or not (n=9-10).

Data were analyzed by unpaired *t* test (D: *L. R*) or Mann-Whitney *U* test (D: *L. M*, *L. I*, *L. J*, E: *E. coli*) and represented as means ± SD. **P* < 0.05, ***P* < 0.01 and *****P* < 0.0001.

**Fig. S3 The level of melatonin in mice with *L. M* colonization.**

(A) Diagram for the description of *L. M* colonization in Abx cocktail pretreated mice.

(B and C) The level of melatonin in the serum (B) and colon (C) of Abx cocktail pretreated mice colonized with *L. M* or not (n=5-9). Melatonin levels in the serum (B) were detected in batches and normalized by external standard.

Data were analyzed by unpaired *t* test (B) or Mann-Whitney *U* test (C) and represented as means ± SD.

**Fig. S4 The level of metabolites in tryptophan-5-HT pathway in culture supernatants of *L. R, L. I, L. J* or *E. coli*.**

(A) Diagram for the description of *L. R*, *L. I* and *L. J* culture supernatant collection at 0 h, 6 h, and 24 h.

(B) The level of tryptophan, 5-HTP, 5-HT, NAS, and melatonin in *L. R* culture supernatants at 0 h, 6 h, and 24 h (n=6).

(C) The level of tryptophan, 5-HTP, 5-HT, NAS, and melatonin in *L. I* culture supernatants at 0 h, 6 h, and 24 h (n=6).

(D) The level of tryptophan, 5-HTP, 5-HT, NAS, and melatonin in *L. J* culture supernatants at 0 h, 6 h, and 24 h (n=6).

(E) Diagram for the description of *E. coli* culture supernatant collection at 0 h and 24 h.

(F) The level of melatonin in *E. coli* culture supernatants at 0 h and 24 h (n=6).

Data were analyzed by one-way ANOVA (B-D) and represented as means ± SD. ***P* < 0.01, ****P* < 0.001 and *****P* < 0.0001.

**Fig. S5 The level of melatonin in mice with *L. R, L. I* or *L. J* culture supernatant gavage.**

(A) Diagram for the description of *L. R*, *L. I*, *L. J* culture supernatant or lactate gavage in mice.

(B) The level of melatonin in the serum of mice treated with culture supernatants of *L. R*, *L. I* or *L. J*, lactate or MRS (n=6-9).

(C) The level of melatonin in the colon of mice treated with *L. R* culture supernatants or MRS (n=9).

Data were analyzed by Kruskal-Wallis (B) or unpaired *t* test (C) and represented as means ± SD. ****P* < 0.001.

**Fig. S6 Establishment of *Myd88*^ΔIEC^ mice.**

(A) Diagram for the strategy of establishment of *Myd88*^ΔIEC^ mice.

(B) Gel electrophoresis of genotype identification of WT, *Myd88^flox/flox^*, *Myd88^flox/flox^* Villin-Cre mice.
