# Supplementary figures and images for: Gut microbiota regulates host melatonin production through epithelial cell MyD88
Source: Gut Microbes. 2024 Feb 14;16(1):2313769. doi: 10.1080/19490976.2024.2313769 (PMC10868534; doi:10.1080/19490976.2024.2313769)

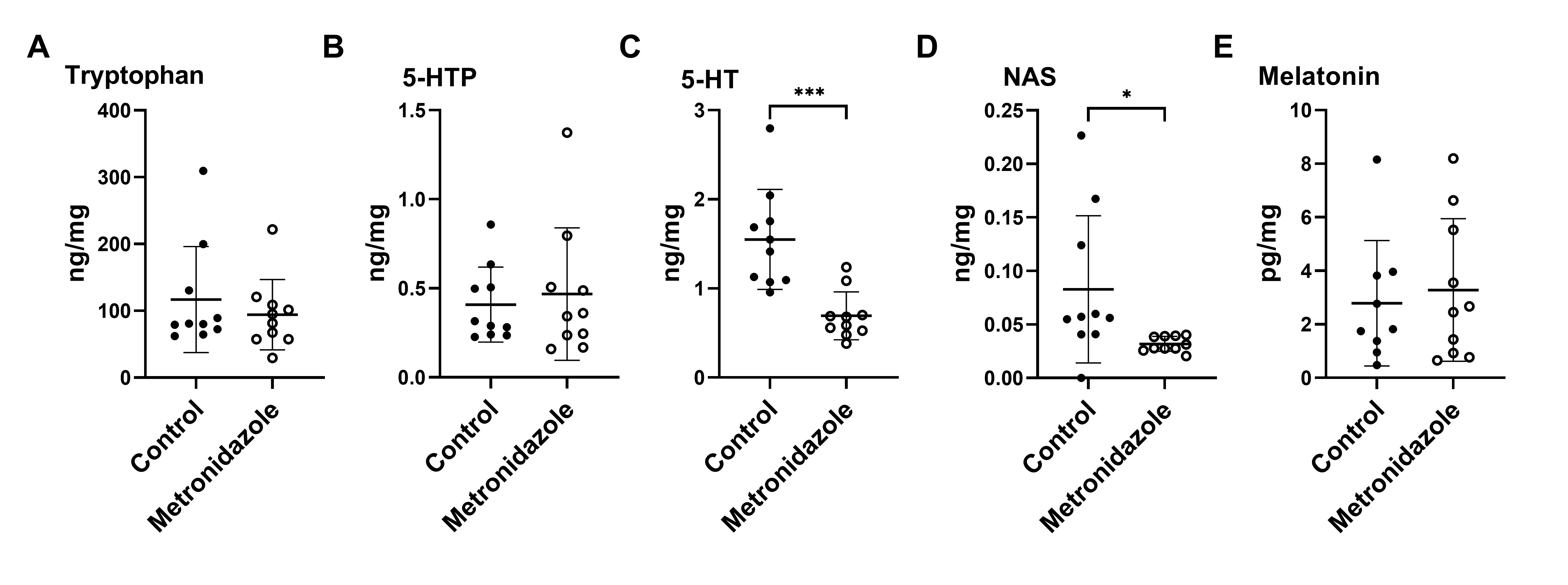

Supplement: Supplemental Material [file KGMI_A_2313769_SM6154.zip › supplementary_files_2313769_1707796552333/Fig S1.tif]

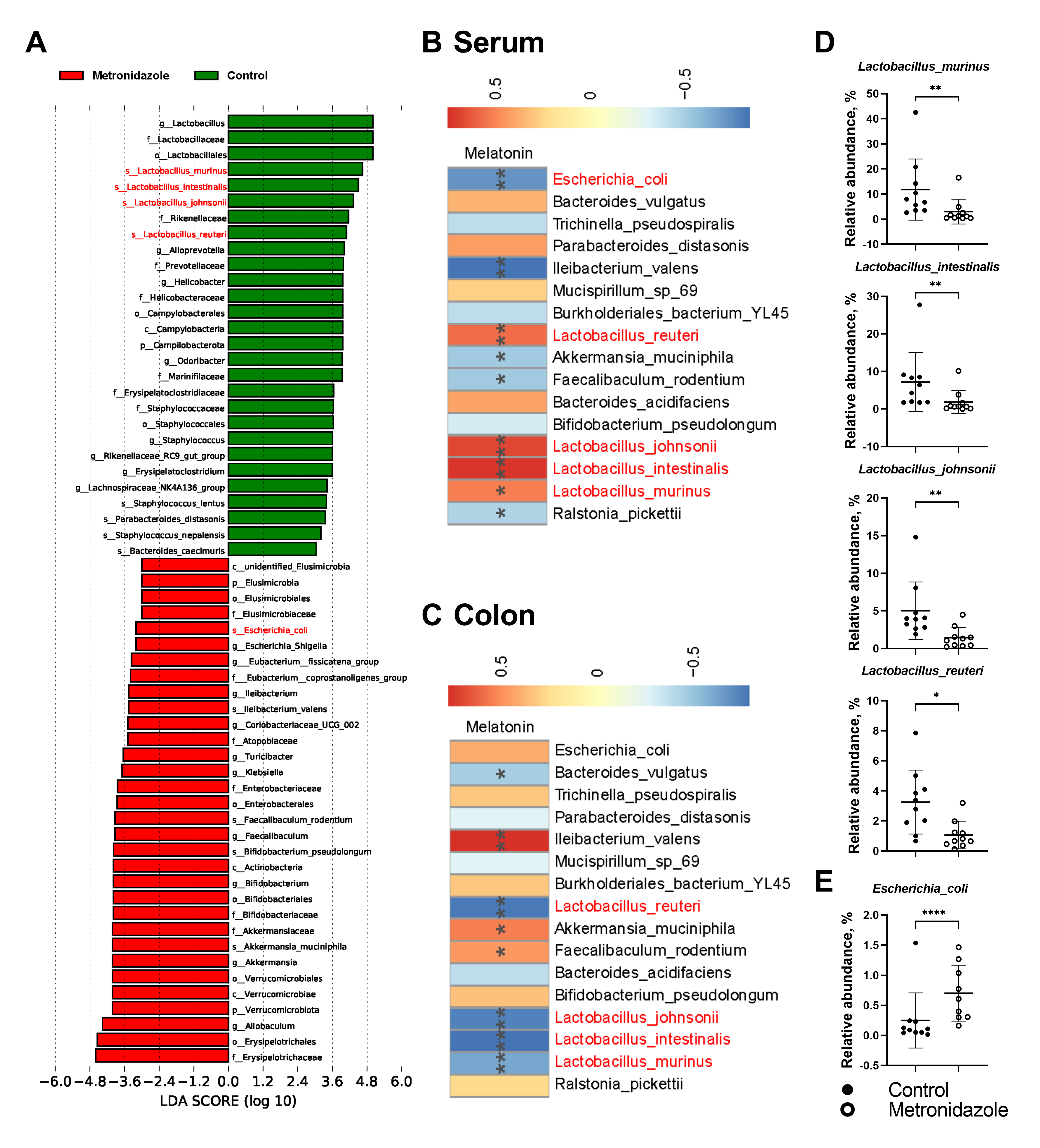

Supplement: Supplemental Material [file KGMI_A_2313769_SM6154.zip › supplementary_files_2313769_1707796552333/Fig S2.tif]

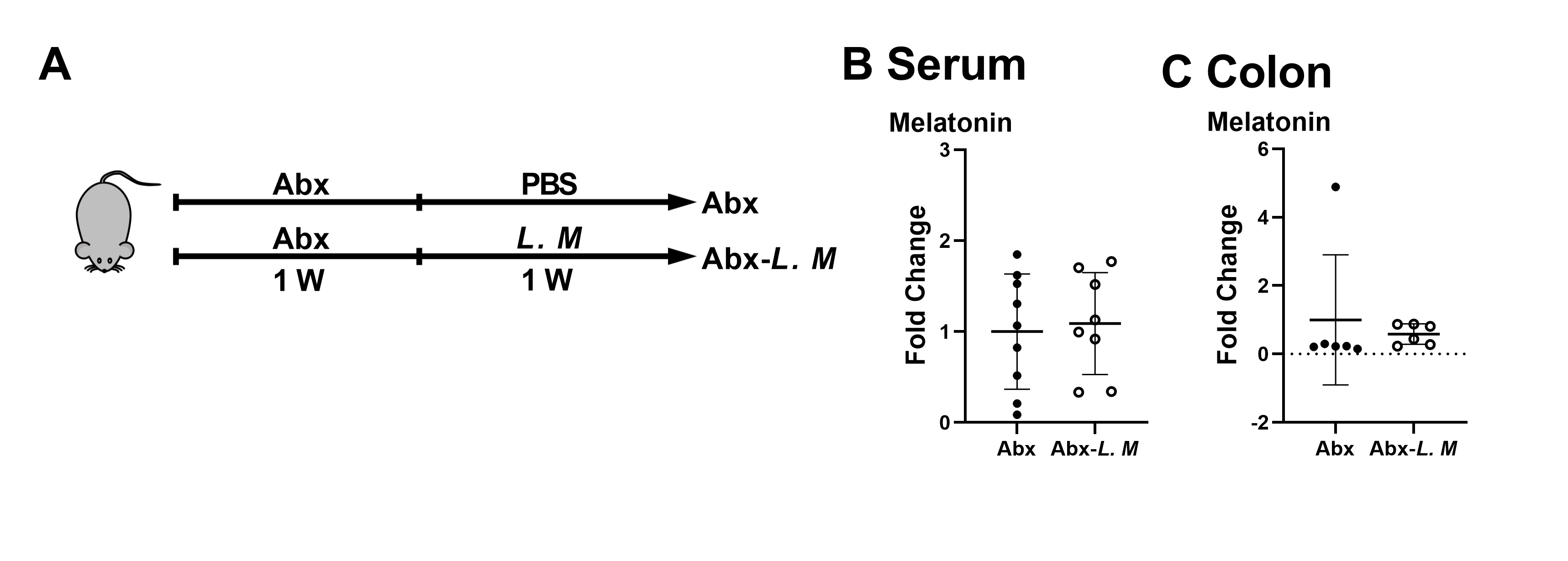

Supplement: Supplemental Material [file KGMI_A_2313769_SM6154.zip › supplementary_files_2313769_1707796552333/Fig S3.tif]

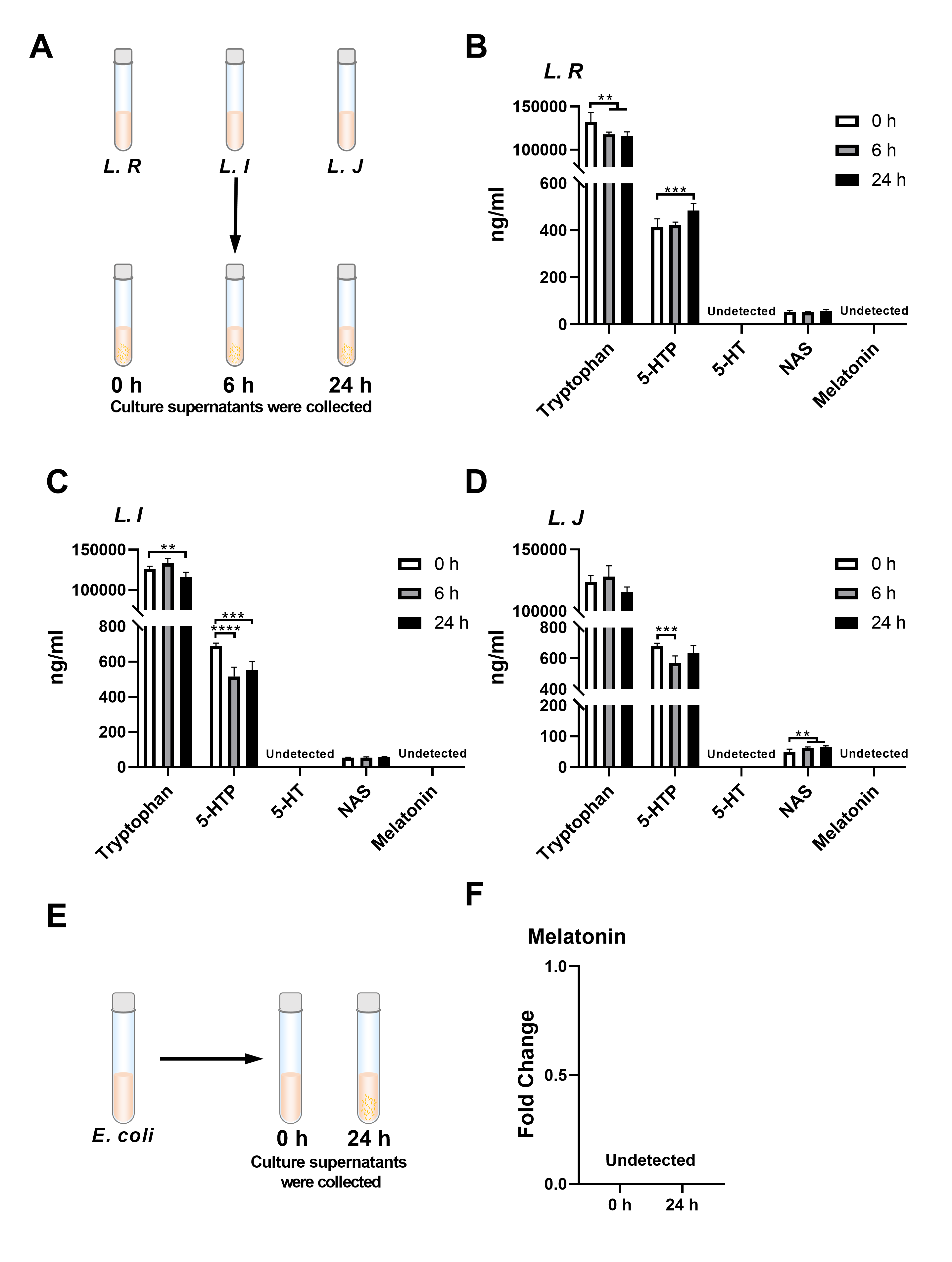

Supplement: Supplemental Material [file KGMI_A_2313769_SM6154.zip › supplementary_files_2313769_1707796552333/Fig S4.tif]

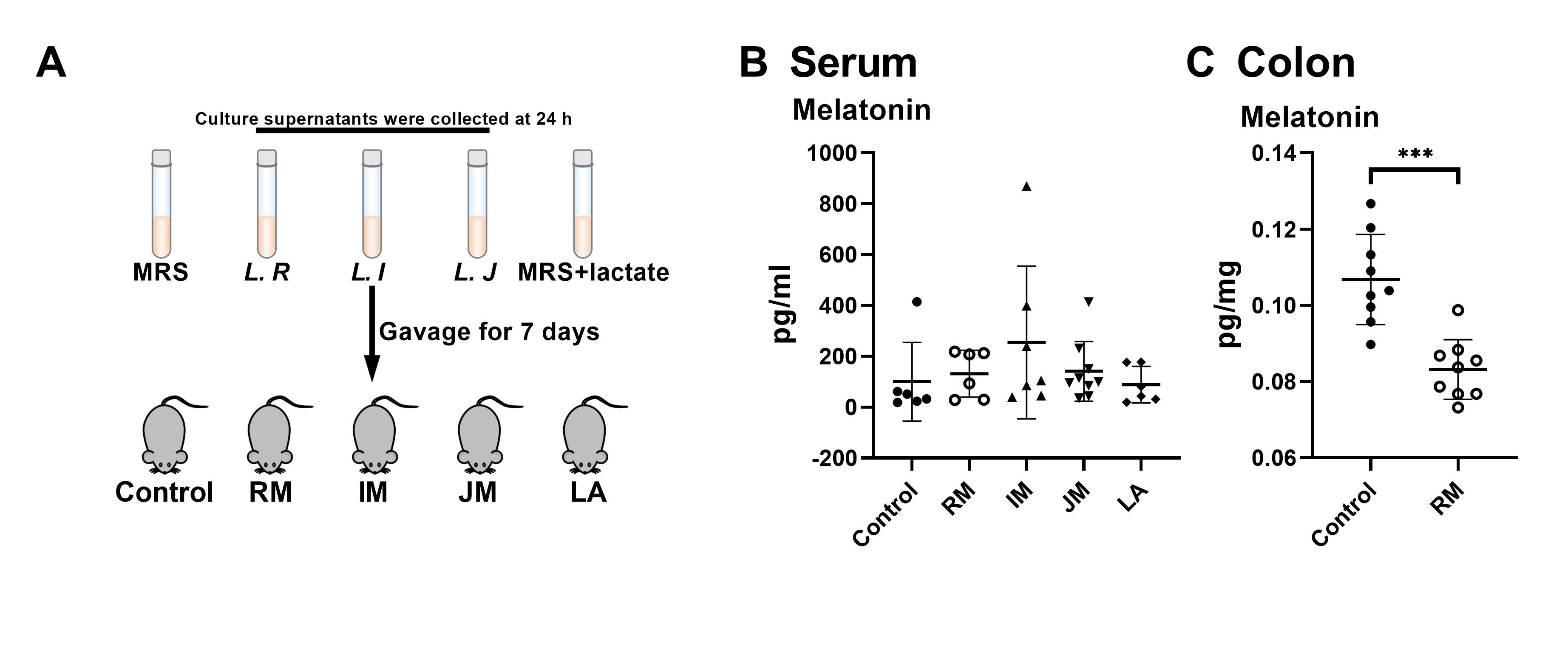

Supplement: Supplemental Material [file KGMI_A_2313769_SM6154.zip › supplementary_files_2313769_1707796552333/Fig S5.tif]

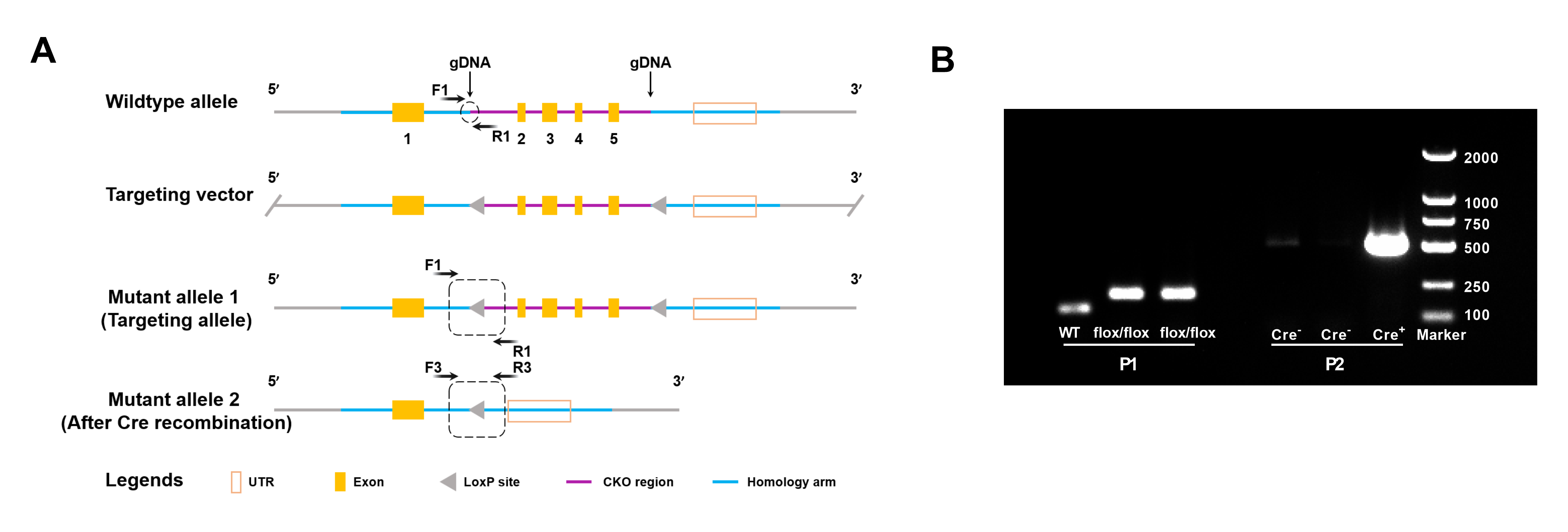

Supplement: Supplemental Material [file KGMI_A_2313769_SM6154.zip › supplementary_files_2313769_1707796552333/Fig S6.tif]
